# Ubiquitination Destabilizes Protein Sphingosine Kinase 2 to Regulate Glioma Malignancy

**DOI:** 10.3389/fncel.2021.660354

**Published:** 2021-07-07

**Authors:** Hongliang Wang, Bing Zhao, Erbao Bian, Gang Zong, Jie He, Yuyang Wang, Chunchun Ma, Jinghai Wan

**Affiliations:** ^1^Department of Neurosurgery, The Second Affiliated Hospital of Anhui Medical University, Hefei, China; ^2^Cerebral Vascular Disease Research Center, Anhui Medical University, Hefei, China; ^3^Department of Neurosurgery, Pudong New Area People’s Hospital, Shanghai, China; ^4^Department of Neurosurgery, National Cancer Center/National Clinical Research Center for Cancer/Cancer Hospital, Chinese Academy of Medical Sciences and Peking Union Medical College, Beijing, China

**Keywords:** glioma, NEDD4L, SPHK2, ubiquitination, posttranslational modification (PTM)

## Abstract

Gliomas are the most common and lethal malignant tumor in the central nervous system. The tumor oncogene sphingosine kinase 2 (SphK2) was previously found to be upregulated in glioma tissues and enhance glioma cell epithelial-to-mesenchymal transition through the AKT/β-catenin pathway. Nevertheless, ubiquitination of SphK2 protein has yet to be well elucidated. In this study, mass spectrometry analysis was performed to identify proteins that interacted with SphK2 protein. Co-immunoprecipitation (co-IP) and immunoblotting (IB) were used to prove the specific interaction between SphK2 protein and the neural precursor cell-expressed developmentally downregulated 4-like (NEDD4L) protein. Fluorescence microscopy was used for detecting the distribution of related proteins. Ubiquitylation assay was utilized to characterize that SphK2 was ubiquitylated by NEDD4L. Cell viability assay, flow cytometry assay, and transwell invasion assay were performed to illustrate the roles of NEDD4L-mediated SphK2 ubiquitination in glioma viability, apoptosis, and invasion, respectively. We found that NEDD4L directly interacted with SphK2 and ubiquinated it for degradation. Ubiquitination of SphK2 mediated by NEDD4L overexpression suppressed glioma cell viability and invasion but promoted glioma apoptosis. Knockdown of NEDD4L presented opposite results. Moreover, further results suggested that ubiquitination of SphK2 regulated glioma malignancy *via* the AKT/β-catenin pathway. *in vivo* assay also supported the above findings. This study reveals that NEDD4L mediates SphK2 ubiquitination to regulate glioma malignancy and may provide some meaningful suggestions for glioma treatment.

## Introduction

Gliomas are the most lethal intracranial tumor with poor prognosis, and there is still no effective therapy up to now (Ouyang et al., 2015). Phospholipids are the frame components of the cell membrane, and the organism is principally constructed of glycerol phospholipids and phosphosphingolipid, whose metabolites such as sphingosine-1-phosphate (S1P) are a key molecule in signaling and play considerably a guiding role in tumors (He et al., 2016). In gliomas, S1P can regulate growth and invasiveness of glioma cells (Young et al., 2009; Bernhart et al., 2015). S1P has also been implicated to promote tumorigenesis and enhance cancer cell proliferation, expansion, and metastasis through the AKT pathway (Beckham et al., 2013; Lee et al., 2017; Tsang et al., 2019).

Sphingosine kinases (SphKs) are the rate-limiting enzymes of S1P and consist of SphK1 and SphK2 (Zhang et al., 2017). In cancers, SphKs could promote cell growth and tumor progression *via* converting the backbone of sphingolipids and sphingosine into S1P (Neubauer and Pitson, 2013; Lee et al., 2015; Neubauer et al., 2016). In gliomas, both SphK1 and SphK2 were abnormally upregulated in tumor tissues and cell lines, and a higher expression of SphKs means lower survival times of glioma patients (Li et al., 2008; Liu et al., 2018). RNA interference-mediated SphK expression inhibition in glioma cells suppressed tumor cell proliferation. Interestingly, knockdown of SphK2 presented more potent inhibition of glioblastoma cell proliferation than SphK1 knockdown (Van Brocklyn et al., 2005). Moreover, SphK2 overexpression enhanced glioma cell epithelial-to-mesenchymal transition through the AKT/β-catenin pathway, a downstream target of S1P (Chen et al., 2019). However, studies on SphK2 inhibitors demonstrated that its low potency, specificity, and selectivity as well as its off-target effects limited its capacity to treat tumors (Hasanifard et al., 2019). Hence, further exploration of the roles and mechanisms of SphK2 in cancers may provide a better suggestion in cancer treatment. Posttranslational modifications like phosphorylation and ubiquitination are important strategies for regulating protein function in cells. Phosphorylation of SphK2 has been fully explored (Hait et al., 2007). Nevertheless, ubiquitination of SphK2 protein is poorly understood. This raises our interests to explore the ubiquitination of SphK2 in gliomas.

Ubiquitination is a posttranslational modification and is involved in regulating diverse cellular processes; for example, it modulates apoptosis and transcription as well as acts as tumor oncogenes or suppressors (Chang and Ding, 2018). In general, ubiquitination levels are in dynamic equilibrium mediated by ubiquitinating enzymes (E1, E2, and E3) and deubiquitinating enzymes (He et al., 2018). The neural precursor cell-expressed developmentally downregulated 4-like (NEDD4L), a member of E3 ubiquitin ligase family, is comprised of an N-terminal C2 domain, a four WW domain, and a C-terminal HECT (homologous to E6-AP C-terminus of the human papilloma virus) ubiquitin-ligase domain. It has been reported that NEDD4L could specifically identify and bind to substrates containing PPXY motifs by its WW domain (Ding et al., 2013). Previously, He et al. reported that NEDD4L was downregulated in glioma tissues and decreased NEDD4L expression was correlated with a worse prognosis of malignant glioma (He et al., 2012).

In this study, we found that NEDD4L interacted with SphK2 and mediated its degradation in a ubiquitination-proteasome-dependent manner. NEDD4L overexpression repressed glioma proliferation and invasion and promoted glioma apoptosis *via* the SphK2/AKT/β-catenin pathway *in vitro* and *in vivo*. This study may provide some suggestions for glioma treatment.

## Materials and Methods

### Cell Culture and Transfection

The human U251 glioma cell lines were purchased from the Chinese Academy of Sciences. The HEK293T cell line was purchased from ATCC. Cells were cultured in Dulbecco’s modified Eagle’s medium (DMEM; Hyclone, Logan, UT, USA) containing 10% fetal bovine serum (FBS; Gibco, Waltham, MA, USA). All cells were cultivated at 37°C in a humidified incubator supplemented with 5% carbon dioxide. Full-length gene sequences of NEDD4L and SphK2 were cloned into pcDNA3.1 vector. HEK293T or glioma cell line was transfected with indicated overexpression plasmids or siRNA with Lipofectamine 2000 (Invitrogen; Thermo Fisher Scientific, Waltham, MA, USA).

### Antibodies

Anti-SphK2 (#32346) and anti-ubiquitin (#3933) antibodies were purchased from Cell Signaling Technology (Danvers, MA, USA). Anti-AKT (ab179463), anti-phospho-AKT (ab38449), and anti-β-catenin (ab16051) antibodies were purchased from Abcam (Cambridge, MA, USA). Anti-β-actin (A1978) antibody was purchased from Sigma (St. Louis, MO, USA). Anti-Flag (RLI-01) and anti-HA (RLI-02) antibodies were purchased from biolinkedin (Shanghai, China).

### Co-immunoprecipitation and immunoblotting

Usually, the transfected cells were harvested after being washed with cold PBS buffer for three times. Cells were scraped into lysis buffer supplemented with protease inhibitor cocktail (Roche, Basel, Switzerland) and then centrifuged at 22,500× *g* for 20 min at 4°C. The related protein expression in the lysates was detected by immunoblotting analysis using indicated antibodies to normalize the total amounts of the inputs. After that, the supernatants were incubated with the indicated antibody and equal amounts of protein A/G beads overnight at 4°C. Finally, the immunocomplexes were pelleted and then resolved in SDS-PAGE for immunoblotting analysis with indicated antibodies.

### Mass Spectrometry Analysis

Samples were prepared using the same IP protocols aforementioned. Samples were separated on SDS-PAGE and subjected to in-gel tryptic digestion, followed by peptide extraction as described before. The extracts were then desalted and concentrated using StageTips, and the eluted peptides were loaded for mass spectrometric analysis. All the sample analyses were performed on nanoscale HLPC-MS system.

### Fluorescence Microscopy

Cells co-transfected with green fluorescent fusion proteins GFP-SphK2 and red fluorescent fusion proteins RFP-NEDD4L were plated on sterile coverslips in six-well plates and cultured for 24 h. Then, the cells were treated with 4% paraformaldehyde, 0.5% Triton X-100, and blocking buffer as well as corresponding primary antibodies in sequence. After that, cells were incubated with the corresponding second antibodies, respectively. Cell nuclear was counterstained with DAPI. Finally, the fluorescence images were captured using Olympus BX51 microscope.

### Ubiquitination Assay

SphK2-Flag was immunoprecipitated from the cells transfected with NEDD4L-HA or vector, and the cell lysates were centrifuged at 22,500× *g* at 4°C for 15 min, and the supernatants were mixed with NEM (N-ethylmaleimide, final concentration 20 mM), followed by SDS-PAGE and immunoblotting analysis with anti-Ub.

### Cell Viability Assay

For the CCK8 assay, the transfected cells were plated into 96-well plates and then cultured for 24 h. After that, the CCK8 solution was added and incubated for 1.5 h. Absorbance at 450 nm was determined using a microplate reader.

### Flow Cytometry Assay

Briefly, for flow cytometry assay, the treated glioma cells were harvested and washed twice in PBS. Annexin V was measured by using Annexin V-FITC/PI Apoptosis Detection Kit (BestBio, Shanghai, China). Flow cytometer (Beckman, Brea, CA, USA) was used to analyze cell apoptotic profile immediately and the CellQuest software was used to analyze the data.

### Transwell Invasion Assay

The transfected glioma cells were plated within the top chamber of 24-well transwell chambers (29017037; Corning, Corning, NY, USA) coated with Matrigel (356234; BD Biosciences, Franklin Lakes, NJ, USA) membrane at a density of 7 × 10^6^ cells/well. The lower chamber was placed with 600 μl DMEM containing 10% FBS. After 48 h of incubation at 37°C, the noninvaded cells in the upper transwell chambers were washed, while the invaded cells were treated with 4% paraformaldehyde and stained with 0.1% crystal violet in sequence. Finally, the invaded cells were counted and photographed at ×100 magnification by using a light microscope (Olympus IX 71, Japan).

### Tumorigenicity Assays in Nude Mice

Glioma U251 cells were transfected with corresponding plasmid and then selected by using puromycin (Sigma). After confirming the expression of the indicated genes, stable cell lines were implanted subcutaneously into 5-week-old female nude mice according to standard procedures. Tumor masses were isolated from the mice for measuring tumor weight 21 days after injection. Expressions of concerned genes were confirmed by immunoblotting analysis with relevant antibodies.

### Statistical Analysis

SPSS 18.0 and GraphPad Prism 6.0 software were sued to analyze experimental results. The data represent mean ± standard error (SEM) and each test was repeated in triplicate at least. When *P*-values < 0.05, it was considered to be statistically significant.

## Results

### NEDD4L Interacts With SphK2

**Figure 1:**
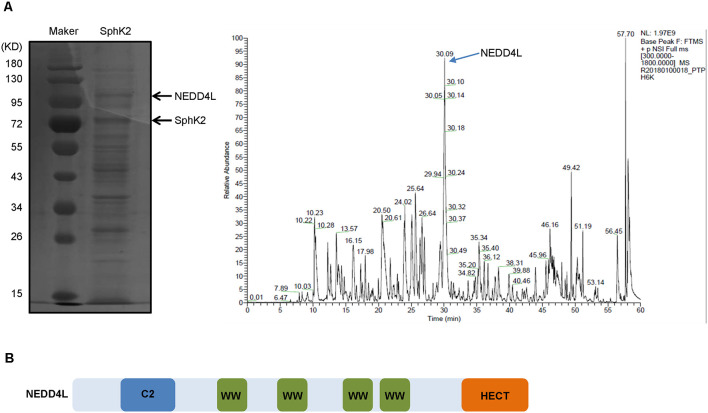
Neural precursor cell-expressed developmentally downregulated 4-like (NEDD4L) gaining the highest interest among sphingosine kinase 2 (SphK2)-associated proteins. **(A)** Glioma U251 cells were transfected with SphK2 overexpression plasmid, and mass spectrometry assay was performed to analyze binding complexes formed with SphK2. The left side of panel **(A)** was a Western blot. The peak marked by the arrow in the right side of panel **(A)** represented NEDD4L. **(B)** Structure model of NEDD4L protein. The WW domain could specifically identify and bind to substrates containing PPXY motifs.

To identify potential modulators of SphK2, we overexpressed SphK2 in glioma U251 cells and performed mass spectrometry analysis of binding complexes formed with SphK2. As shown in Figure 1A, several bands of potential interest were observed, and among the identified SphK2-associated proteins, NEDD4L, a ubiquitin E3 ligase, was the most interesting. NEDD4L has been reported to specifically identify and bind to substrates containing PPXY motifs by its WW domain (Ding et al., 2013). To our delight, NEDD4L possesses a four WW domain and the SphK2 protein contains a PPXY domain, which suggested that NEDD4L might interact with SphK2 (Figure 1B).

**Figure 2:**
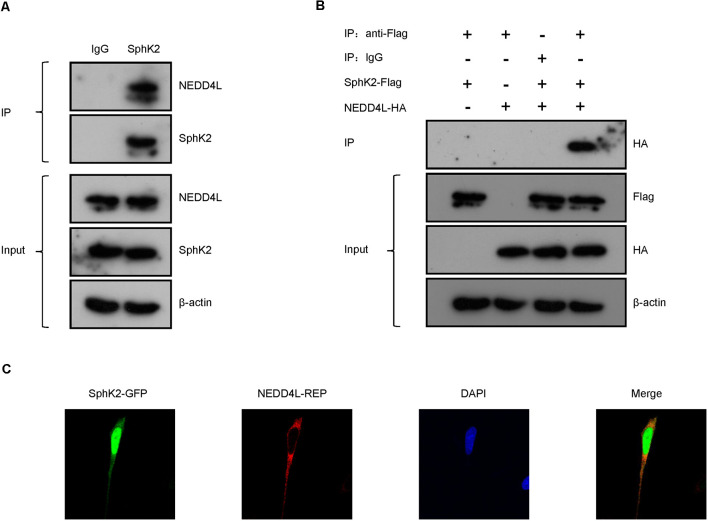
NEDD4L interacts with SphK2. **(A)** Anti-SphK2 was used to precipitate the protein complexes in HEK293T cells and anti-NEDD4L was used to perform co-IP assay. **(B)** HEK293T cells were transfected with SphK2-Flag and/or NEDD4L-HA overexpression plasmid. Anti-Flag was used to precipitate the protein complexes and anti-HA was used to perform the co-IP assay. **(C)** Fluorescence co-localization assay was used to confirm that NEDD4L was co-expressed with SphK2 in the cytoplasm in HEK293T cells.

To confirm the interaction between NEDD4L and SphK2, SphK2 was immunoprecipitated from the cultured HEK293T cells and NEDD4L was detected by immunoblotting. As shown in Figure 2A, SphK2 was observed to interact with endogenous NEDD4L. Then, HEK293T cell was transfected with Flag-tagged SphK2 and/or HA-tagged NEDD4L and exogenous co-immunoprecipitation (co-IP) assay was performed. SphK2 was immunoprecipitated from the cultured HEK293T cells by using Flag beads and then HA-tagged NEDD4L was detected by immunoblotting. As shown in Figure 2B, we found that NEDD4L interacted with SphK2. In addition, fluorescence co-localization assay indicated that NEDD4L was co-expressed with SphK2 in the cytoplasm, which further supported the above findings (Figure 2C). These results suggested that NEDD4L could interact with SphK2.

### NEDD4L Mediates the Degradation of SphK2 Protein Through the Ubiquitin-Proteasomal Pathway

**Figure 3:**
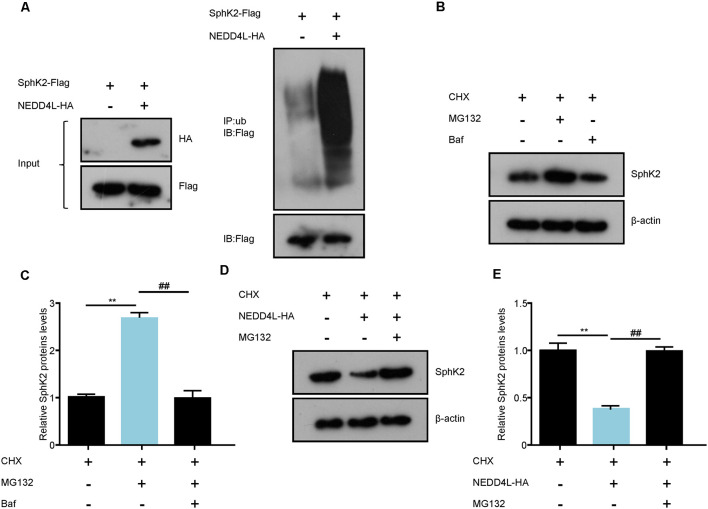
NEDD4L ubiquitinates SphK2 protein and mediates its degradation. **(A)** HEK293T cells were transfected with SphK2-Flag and/or NEDD4L-HA overexpression plasmid and then SphK2 protein was immunoprecipitated by anti-Flag to detect its ubiquitination level. **(B,C)** BAF or MG132 treated HEK293T cells to explore the way how the SphK2 protein degraded. **P < 0.01 compared with cycloheximide (CHX); ^##^*P* < 0.01 compared with CHX in combination with BAF. **(D,E)** HEK293T cells were transfected with NEDD4L-HA overexpression plasmid and/or MG132 and the levels of SphK2 protein were detected. **P < 0.01 compared with CHX; ^##^*P* < 0.01 compared with CHX in combination with NEDD4L overexpression.

The above findings have driven us to perform a ubiquitination assay to assess whether the E3 ubiquitin ligase NEDD4L modulated the posttranscriptional activity of SphK2. Flag-tagged SphK2 and/or HA-tagged NEDD4L was stably transfected with HEK293T cell. Then, the SphK2 protein was immunoprecipitated to detect its ubiquitination level. As shown in Figure 3A, NEDD4L overexpression significantly increased the ubiquitination levels of SphK2 compared with the control. This indicated that NEDD4L E3 ubiquitin ligase was important for regulating SphK2 ubiquitination.

Protein ubiquitination usually mediates the degradation of proteins into the proteasomal pathway. Next, we investigated whether NEDD4L could regulate SphK2 protein degradation. Generally, proteins undergo degradation through the autophagy-lysosomal pathway or the ubiquitin-proteasomal pathway. First, we treated HEK293T cells with BAF, an autophagy inhibitor, and MG132, a proteasome inhibitor, respectively, and then detected SphK2 protein levels by immunoblotting. As shown in Figures 3B,C, SphK2 protein levels were increased with MG132 treatment compared with the cycloheximide (CHX) control, while cells treated with BAF almost did not change on SphK2 protein levels. This means that the SphK2 protein undergoes degradation through ubiquitination-proteasome systems. Further results showed that overexpression of NEDD4L apparently decreased SphK2 protein levels, while this could be reversed by MG132 (Figures 3D,E). Taken together, these results strongly demonstrated that NEED4L functioned as an E3 ligase to mediate the ubiquitination and degradation of SphK2.

### Ubiquitination of SphK2 Suppresses Glioma Proliferation and Invasion and Promotes Glioma Apoptosis *In vitro*

**Figure 4:**
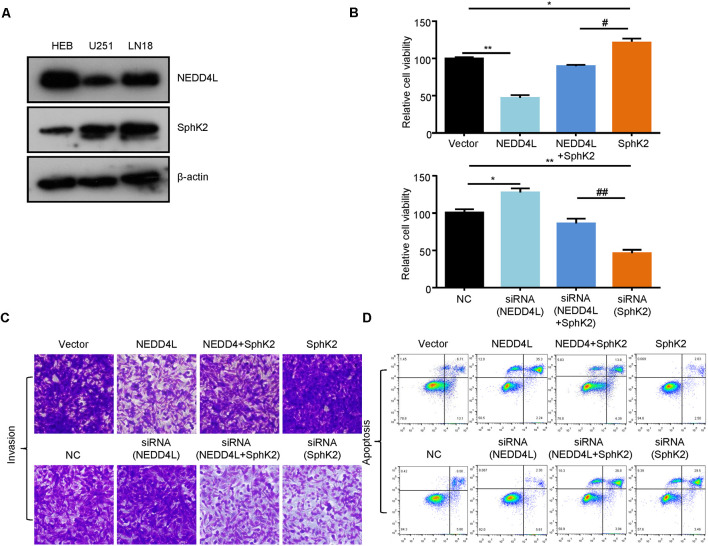
Ubiquitination of SphK2 suppresses glioma cell progression *in vitro*. **(A)** The expression levels of SphK2 and NEDD4L in normal brain cells and glioma cells. **(B–D)** Glioma U251 cells were transfected with NEDD4L and/or SphK2 overexpression plasmid or transfected with NEDD4L and/or SphK2 siRNA, and cell viability, apoptosis, and invasion were measured by the CCK8 assay, invasion assay, and flow cytometry assay in sequence. *P < 0.05, **P < 0.01 compared with the vector or NC; ^#^*P* < 0.05, ^##^*P* < 0.01 compared with SphK2 overexpression plasmid or siRNA.

**Figure 5:**
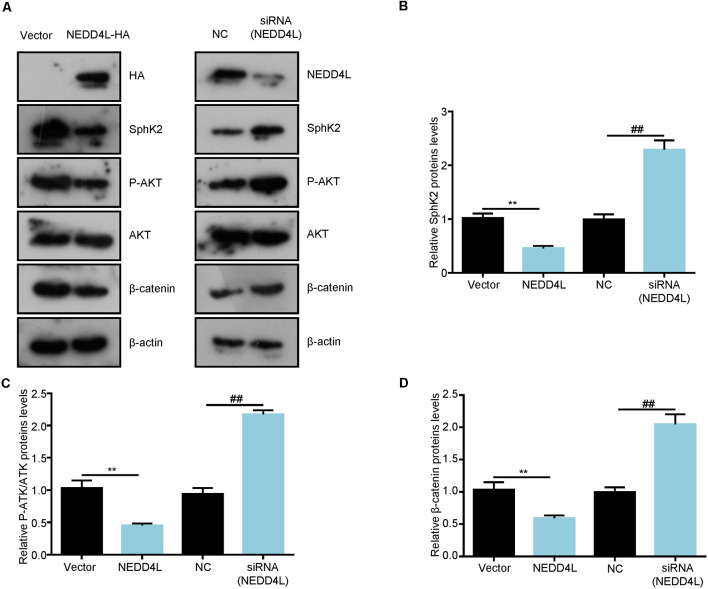
Ubiquitination of SphK2 inhibits glioma cell progression through the AKT/β-catenin pathway. **(A–D)** Glioma U251 cells were transfected with NEDD4L overexpression plasmid or siRNA and indicated proteins were detected by immunoblotting (IB). **P < 0.01 compared with the vector; ^##^*P* < 0.01 compared with NC.

SphK2 has previously been reported to be upregulated in glioma tissues and played as an oncogene (Chen et al., 2019). Thus, we speculated that NEDD4L-mediated ubiquitination of the SphK2 protein might reverse its role in gliomas. First, we found that NEDD4L expression levels were decreased in glioma cells compared with normal brain cells, whereas SphK2 expression was the opposite (Figure 4A). As shown in Figures 4B,C, compared with the vector, overexpression of NEDD4L suppressed glioma U251 cell viability and invasion, while SphK2 overexpression promoted glioma cell viability and invasion. Compared with SphK2 overexpression alone, in combination with NEDD4L and SphK2, overexpression reversed SphK2-mediated promotion of glioma viability and invasion. Knockdown of both NEDD4L and SphK2 did the opposite result. The results of flow cytometry assay also showed that NEDD4L abolished the inhibition of glioma cell apoptosis and death mediated by SphK2 (Figure 4D). In addition, NEDD4L overexpression induced SphK2 ubiquitination, which in turn inhibited the AKT/β-catenin pathway, and knockdown of NEDD4L did the opposite results (Figure 5). These results indicated that NEDD4L-mediated SphK2 ubiquitination negatively regulated glioma cell malignancy *via* the AKT/β-catenin pathway.

### Ubiquitination of SphK2 Functions as a Glioma Suppressor *In vivo*

**Figure 6:**
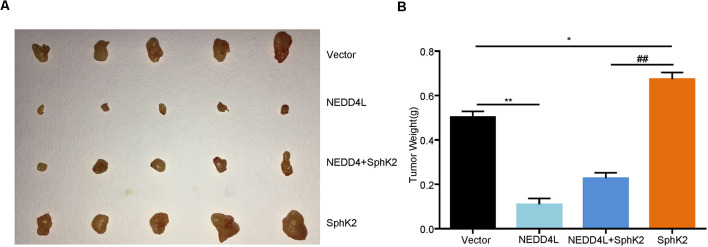
Ubiquitination of SphK2 inhibits glioma growth *in vivo*. **(A,B)** Glioma U251 cells stably overexpressed of NEDD4L and/or SphK2 were implanted into 5-week-old female nude mice, and tumor weight was measured 21 days after injection. *P < 0.05, **P < 0.01 compared with the vector; ^##^*P* < 0.01 compared with SphK2 overexpression plasmid.

**Figure 7:**
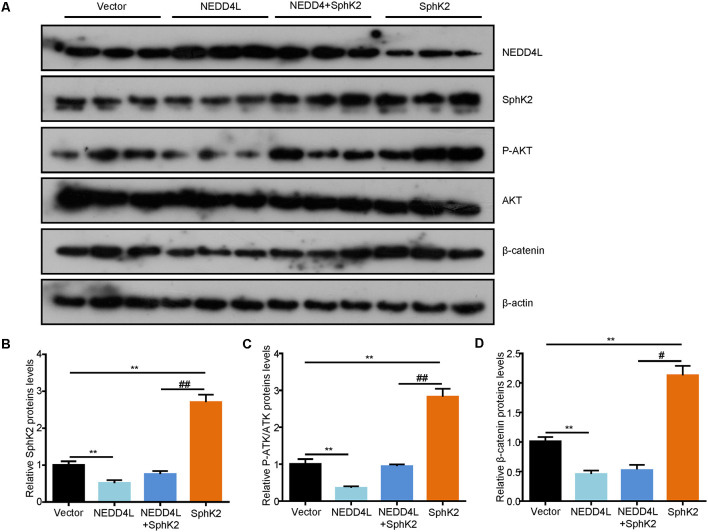
Ubiquitination of SphK2 suppresses glioma growth *via* AKT/β-catenin pathway *in vivo*. **(A,B)** Total proteins were isolated from tumor tissues and expressions of concerned genes were confirmed by IB analysis with relevant antibodies. **(C)** Relative protein levels of P-AKT.**(D)** Relative protein levels of β-catenin. **P < 0.01 compared with the vector; ^#^*P* < 0.05, ^##^*P* < 0.01 compared with SphK2 overexpression plasmid.

After that, to further investigate the role of ubiquitination of SphK2 *in vivo*, a xenograft tumor-bearing model was established by inoculating NEDD4L overexpression plasmid and/or SphK2 overexpression plasmid transfected U251 into nude mice. As shown in Figures 6A,B, overexpression of SphK2 significantly promoted glioma growth, and this could be partially reversed following NEDD4L in combination with SphK2 overexpression. Moreover, NEDD4L-mediated SphK2 ubiquitination apparently decreased the levels of the SphK2 protein, phosphorylation of the AKT protein, and β-catenin protein levels (Figure 7). These results revealed that ubiquitination of SphK2 functioned as a tumor suppressor through inhibiting the AKT/β-catenin pathway *in vivo*.

## Discussion

**Figure 8:**
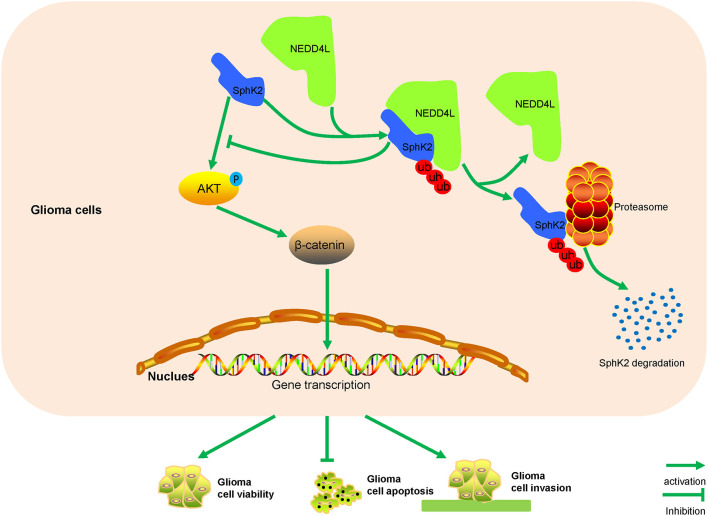
A model for how NEDD4L-mediated SphK2 ubiquitination performs its roles in gliomas. NEDD4L targets SphK2 protein for degradation in the ubiquitin-proteasomal pathway, thus inhibiting the AKT/β-catenin pathway to regulate glioma proliferation, apoptosis, and invasion.

Gliomas are the most common and lethal malignant tumor in the central nervous system, and glioma patients generally have a poor prognosis (Bao et al., 2006). There are still no special effective therapeutics for glioblastomas despite surgery, radiotherapy, and chemotherapy treatment (Pisati et al., 2007). Considering a high degree of malignancy and unfavorable prognosis of gliomas, new therapeutic targets are urgently needed. In the present study, we reported that NEDD4L interacted with SphK2 to mediate its degradation in a ubiquitin-proteasomal manner. The ubiquitination of SphK2 mediated by NEDD4L negatively regulated glioma malignancy *via* the AKT/β-catenin pathway (Figure 8).

SphKs consist of two distinct isoforms, SphK1 and SphK2, are highly conserved enzymes found in diverse organisms like mammals and plants, and catalyze the phosphorylation of sphingosine to generate S1P (Hait et al., 2005). SphKs were found abnormally expressed in several tumor types and had an oncogenic role (Sankala et al., 2007; Venkata et al., 2014; Wallington-Beddoe et al., 2014; Tsang et al., 2019; Sukocheva et al., 2020). In gliomas, SphK1 was reported to be significantly correlated with the histologic grade of tumor, and patients with high SphK1 levels generally exhibited a poor survival time (Li et al., 2008). In glioma cells, SphK1 could promote tumor cell proliferation and invasion, but inhibit cell apoptosis through the AKT pathway activation (Radeff-Huang et al., 2007; Kapitonov et al., 2009; Young et al., 2009; Guan et al., 2011). Recently, Liu et al. reported that SphK2 was increased in glioma tissues than in normal brain tissues and positively correlated with glioma proliferation (Liu et al., 2018). Similar to SphK1, SphK2 also targeted the AKT pathway to regulate glioma cell proliferation and EMT (Chen et al., 2019). Interestingly, knockdown of SphK2 presented more potent inhibition of glioblastoma cell proliferation than SphK1 knockdown (Van Brocklyn et al., 2005). Hence, we chose SphK2 in this study.

Due to the oncogenic roles of SphK2 in tumors, studies focused on SphK2 inhibitors have been reported. However, the low potency, specificity, and selectivity as well as the off-target effects of SphK2 inhibitors limited its capacity to treat tumors (Hasanifard et al., 2019). Thus, further exploration of the roles and mechanisms of SphK2 in cancers may provide a better suggestion in cancer treatment. Generally, transcription of SphK2 was promoted by activated CREB directly binding to the promoter region of SphK2. The catalytic activity of SphK2 could be stimulated by interleukin-1β, TNF-α, epidermal growth factor, and hypoxia-inducible factor 1α (Hait et al., 2005; Mastrandrea et al., 2005; Schnitzer et al., 2009; Mizutani et al., 2015). Nevertheless, the ubiquitination of SphK2 is poorly studied. In this study, we overexpressed SphK2 in glioma cells and performed mass spectrometry to analyze the binding complexes formed with SphK2. NEDD4L was the most attractive protein among several proteins of interest. The SphK2 protein contains a PPXY domain and NEDD4L possesses a four WW domain, which has been reported to specifically identify and bind to substrates containing PPXY motifs (Ding et al., 2013). To our delight, both endogenous and exogenous co-IP assays suggested that NEDD4L directly interacted with SphK2. In addition, fluorescence co-localization assay was in agreement with the above findings. However, in this study, we just proved that NEDD4L could interact with SphK2, and the exact binding site has not been explored, which is the field we will focus on in the subsequent study. Apart from that, emerging research reported that phosphorylation of proteins at multiple Ser/Thr sites could preclude its ubiquitination. For example, Nanog protein is phosphorylated at multiple Ser/Thr-Pro motifs, which leads to Nanog stabilization by suppressing its ubiquitination (Moretto-Zita et al., 2010). Enzymatic analysis showed that PTEN Ser/Thr phosphorylation inhibited PTEN ubiquitylation by WWP2 (Chen et al., 2016). A previous study revealed that ERK1/2 phosphorylates SphK2 protein at Ser387 and Thr614 on a long variant of SphK2 and Ser351 and/or Thr578 on the short isoform of SphK2 (Hait et al., 2007). However, whether phosphorylation of SphK2 could affect its ubiquitination needs further exploration.

NEDD4L is a member of E3 ubiquitin ligase family and is known to bind and regulate various membrane proteins to promote their internalization and turnover (Goel et al., 2015). Nazio found that NEDD4L could specifically ubiquitinate ULK1 kinase (Nazio and Cecconi, 2017). NEDD4L was also reported to ubiquitinate CREB-regulated transcription coactivator 3, thus limiting cAMP signaling (Kim et al., 2018). In this study, we found that NEDD4L interacted with SphK2 to ubiquitinate SphK2 protein. It was widely accepted that proteins usually undergo degradation through the autophagy-lysosomal pathway or the ubiquitin-proteasomal pathway. Thus, we hypothesized that NEDD4L might mediate SphK2 protein degradation in a ubiquitin-proteasomal manner. Then, we treated HEK293T cells with the ubiquitination-proteasome inhibitor MG132 and the autophagy inhibitor BAF, respectively. We found that MG132 protected the SphK2 protein from degradation, which indicated that SphK2 was degraded *via* ubiquitination-proteasome systems. Besides, NEDD4L overexpression decreased the levels of SphK2 protein and this could be abolished by MG132. Taken together, we concluded that NEDD4L functioning as an E3 ligase could target SphK2 for degradation in a ubiquitin-proteasomal manner.

Ubiquitination of SphK2 decreased its protein levels, which might have an effect on the roles of SphK2 in glioma. Our subsequent results showed that overexpressed SphK2 played as an oncogene, which was consistent with previous reports (Neubauer et al., 2016; Chen et al., 2019). Moreover, NEDD4L overexpression induced the ubiquitination of SphK2 reversing the promotion of glioma cells mediated by SphK2. In addition, it was reported that SphK2 regulated tumor malignancy *via* modulating the AKT/β-catenin pathway (Chen et al., 2019). Our results also showed that ubiquitination of SphK2 negatively regulated glioma malignancy through suppressing the AKT/β-catenin pathway. Further *in vivo* assay also confirmed these findings. However, previous studies reported that SphK2 is the rate-limiting enzyme of S1P and S1P plays its role *via* activation of oncogenic Akt signaling (Beckham et al., 2013; Lee et al., 2017). Hence, our results indicated that ubiquitination of SphK2 regulated glioma malignancy *via* indirectly targeting the AKT/β-catenin pathway.

A recent study pointed out that downregulation of NEDD4L promoted hepatocellular carcinoma growth (Zhao et al., 2018). Moreover, NEDD4L overexpression suppressed tumor cell proliferation, migration, and invasion in nonsmall cell lung cancer (Wang et al., 2019). Downregulation of NEDD4L was also reported in glioma tissues (He et al., 2012). In the present study, we showed that NEDD4L overexpression inhibited glioma cell viability and invasion. Opposite results were presented when NEDD4L was knocked down. Overexpression of NEDD4L promoted glioma apoptosis, while NEDD4L knockdown inhibited glioma apoptosis. Furthermore, NEDD4L overexpression-mediated SphK2 ubiquitination suppressed the AKT/β-catenin pathway and NEDD4L knockdown did the opposite. These results showed that NEDD4L might play its role in glioma *via* targeting the SphK2/AKT/β-catenin pathway. Taken together, all these results supported our findings that NEDD4L-mediated SphK2 ubiquitination functioned as a tumor suppressor in gliomas.

## Data Availability Statement

The original contributions presented in the study are included in the article, further inquiries can be directed to the corresponding author.

## Ethics Statement

The animal study was reviewed and approved by Ethics Committee of the Second Affiliated Hospital of Anhui Medical University.

## Author Contributions

JW and HW designed research, HW, BZ, and GZ performed the experiments. JH and YW analyzed the data. BZ and CM supervised the study. HW wrote the manuscript. All authors contributed to the article and approved the submitted version.

## Conflict of Interest

The authors declare that the research was conducted in the absence of any commercial or financial relationships that could be construed as a potential conflict of interest.

## Abbreviations

SphK2: sphingosine kinase 2
SphKs: sphingosine kinases
NEDD4L: neural precursor cell-expressed developmentally downregulated 4-like
S1P: sphingosine-1-phosphate
HECT: homologous to E6-AP C-terminus of the human papilloma virus
BAF: bafilomycin A1.
